# Source-stratified gut–extraintestinal organ crosstalk in sepsis-associated acute gastrointestinal injury and paralytic ileus: the gut as both driver and target

**DOI:** 10.3389/fmed.2026.1831340

**Published:** 2026-04-13

**Authors:** Congcong Qin, Weiwei Wang, Qinyuan Du, Li Kong, Guochen Li

**Affiliations:** 1 Institute of Chinese Medical Literature and Culture, Shandong University of Traditional Chinese Medicine, Jinan, Shandong, China; 2 The First Clinical Medical College, Shandong University of Traditional Chinese Medicine, Jinan, Shandong, China; 3 Affiliated Hospital of Shandong University of Traditional Chinese Medicine, Jinan, Shandong, China

**Keywords:** acute gastrointestinal injury, extracellular vesicles, organ crosstalk, paralytic ileus, sepsis

## Abstract

Sepsis-associated acute gastrointestinal injury and paralytic ileus are common but underrecognized manifestations of systemic critical illness and are closely linked to feeding intolerance, barrier failure, secondary infection, and progression to multiorgan dysfunction. However, the gut in sepsis should not be viewed solely as a passive target of distant organ injury. Owing to its unique microbial burden, highly specialized epithelial–immune barrier, and central role in host–microbe and metabolic homeostasis, the gut may function either as an initiator of injury amplification or as a vulnerable downstream target, depending on the infectious source and disease stage. In this review, we propose a source-stratified framework for gut–extraintestinal organ crosstalk in sepsis-associated acute gastrointestinal injury and paralytic ileus. In enterogenic sepsis, the gut more commonly serves as an injury driver through barrier disruption, microbial translocation, dysbiosis, and propagation of inflammatory and metabolic stress signals. In extraintestinal sepsis, the gut more often emerges as a susceptible target of systemic inflammation, microcirculatory failure, neurohumoral dysregulation, and organ-to-organ injury transmission, while subsequent gut dysfunction may in turn amplify remote organ damage. These distinct starting points ultimately converge into a self-reinforcing loop involving epithelial and endothelial barrier failure, immune dysregulation, mitochondrial dysfunction, immunometabolic reprogramming, regulated cell death, extracellular vesicle-mediated signaling, and bidirectional organ injury amplification. We further summarize how these shared mechanisms shape the gut–lung, gut–brain, gut–liver, gut–kidney, and gut–heart axes, and discuss their implications for biomarker development, bedside phenotyping, source-based risk stratification, and mechanism-guided therapeutic strategies. By reframing the gut as both driver and target within a source-dependent network of organ crosstalk, this review aims to provide a more integrative pathobiological model for sepsis-associated gastrointestinal dysfunction and to inform future translational and clinical studies.

## 1 Introduction

Sepsis remains a leading cause of death and prolonged intensive care unit stay worldwide, not only because of uncontrolled infection but also because dysregulated host responses propagate tissue injury across organs (1, 2). Within this systemic process, gastrointestinal dysfunction is increasingly recognized as a major determinant of disease progression rather than a secondary epiphenomenon (3). Sepsis-associated acute gastrointestinal injury and paralytic ileus are common in critically ill patients and are closely linked to impaired enteral nutrition delivery, bacterial and endotoxin translocation, abdominal complications, and adverse outcomes (4). In this review, sepsis-associated acute gastrointestinal injury is used as the broader clinical framework, whereas paralytic ileus is discussed as a severe motility-dominant manifestation within this spectrum (3). Yet despite their clinical relevance, gastrointestinal manifestations in sepsis are still often discussed mainly in terms of feeding intolerance or supportive care, while their broader role in multiorgan pathobiology remains insufficiently conceptualized (4).

The gut differs fundamentally from many other organs involved in sepsis-related crosstalk (5). It contains the body’s largest microbial reservoir, a highly specialized epithelial barrier, and dense immune and neurohumoral regulatory networks that are essential for systemic homeostasis (6). Once disrupted, this barrier–microbiota–immune interface can shift from a homeostatic regulator to a source of inflammatory amplification, microbial dissemination, vascular dysfunction, and distal organ injury (6). At the same time, the gut is highly vulnerable to hypoperfusion, venous congestion, endothelial dysfunction, autonomic imbalance, and inflammatory injury arising from remote organ failure (7). Previous organ-crosstalk reviews have provided an important conceptual basis for understanding how sepsis-related injury propagates across the lung and extrapulmonary organs (8). However, the gut differs fundamentally from the lung in that it serves not only as a vulnerable target of systemic injury but also as a major microbial reservoir, barrier organ, and potential initiator of outward injury propagation (7, 8). Current narratives therefore remain insufficient if they do not distinguish the gut according to infectious source and initial pathobiological context (8).

**Figure 1:**
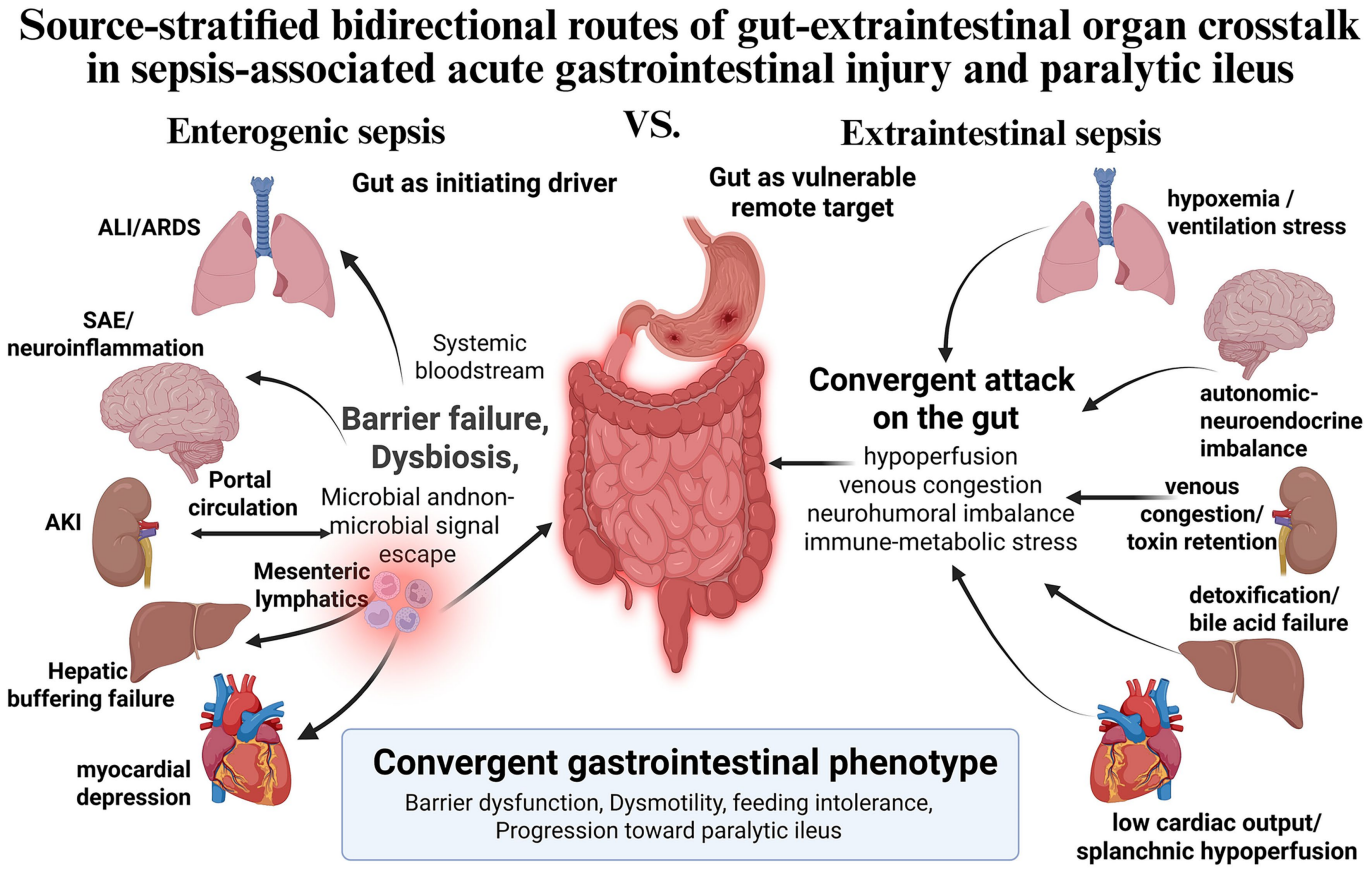
Source-stratified bidirectional routes of gut–extraintestinal organ crosstalk in sepsis-associated acute gastrointestinal injury and paralytic ileus. This figure illustrates the overarching source-stratified conceptual framework of the review. In enterogenic sepsis, the gut more often acts as an initiating driver of outward injury propagation through barrier failure, dysbiosis, and the escape of microbial and non-microbial injurious signals via the portal circulation, mesenteric lymphatics, and systemic bloodstream, thereby contributing to secondary injury in the lung, brain, kidney, liver, and heart. In extraintestinal sepsis, by contrast, the gut more often emerges as a vulnerable remote target of extraintestinal organ-derived insults, including hypoxemia and ventilation stress, autonomic-neuroendocrine imbalance, venous congestion and toxin retention, detoxification and bile acid failure, and low cardiac output with splanchnic hypoperfusion. Despite these distinct starting points, both routes ultimately converge on a common gastrointestinal phenotype characterized by barrier dysfunction, dysmotility, feeding intolerance, and progression toward paralytic ileus. ALI, acute lung injury; ARDS, acute respiratory distress syndrome; SAE, sepsis-associated encephalopathy; AKI, acute kidney injury.

Based on this perspective, the present review proposes a gut-centered, source-stratified framework for sepsis-associated acute gastrointestinal injury and paralytic ileus (9). In enterogenic sepsis, the gut more often acts as an initiating driver of outward injury propagation through barrier collapse, microbial overgrowth, mesenteric immune activation, and translocation of harmful luminal products (10). In extraintestinal sepsis, by contrast, the gut more often emerges as a vulnerable remote target of systemic inflammation, microcirculatory dysfunction, organ-to-organ injury transfer, and neuroendocrine stress, while secondary intestinal dysfunction may further accelerate multiorgan deterioration (11). Although these distinct starting points arise from different pathobiological contexts, they may ultimately converge into a shared gastrointestinal phenotype characterized by barrier dysfunction, dysmotility, enteral intolerance, and progression toward paralytic ileus (12). We therefore first summarize the shared mechanisms underlying bidirectional injury propagation and then discuss the directional gut–lung, gut–brain, gut–liver, gut–kidney, and gut–heart axes, with emphasis on how distinct infectious sources shape the initiation and amplification of organ injury (11, 12). Unlike a lung-centered framework, a gut-centered view of sepsis must account simultaneously for the intestine as a microbial reservoir, a barrier organ, and a potential amplifier of outward injury propagation. These source-stratified bidirectional routes and their convergence into a common gastrointestinal phenotype are schematically illustrated in Figure 1.

## 2 Why the gut occupies a distinct position in source-stratified organ crosstalk

### 2.1 Barrier failure and permeability remodeling

Barrier failure and permeability remodeling constitute the structural foundation of gut–extraintestinal organ crosstalk in sepsis-associated acute gastrointestinal injury and paralytic ileus (5). Unlike many other organs, the gut forms a uniquely exposed interface between the host internal milieu and a dense luminal microbial ecosystem. This interface depends on the continuous integrity of epithelial renewal, tight-junction organization, mucosal immune surveillance, enteric neural regulation, and gut vascular perfusion (13). During sepsis, the villus tip is particularly vulnerable to hypoperfusion and hypoxia, the intestinal epithelium faces high energetic demands because of rapid cellular turnover, and tight junctions are readily disrupted by inflammatory and oxidative stress. For these reasons, the gut frequently develops barrier instability, permeability increase, mucosal edema, and motility suppression at an early stage, thereby assuming a position that is both highly vulnerable to systemic injury and highly capable of redistributing local injury signals outward (14).

Importantly, barrier failure in sepsis should not be reduced to a nonspecific consequence of global inflammation. Although inflammatory mediators circulate systemically, organ injury is not distributed in a truly uniform manner. Hemodynamic characteristics, endothelial phenotypes, adhesion molecule expression, local chemotactic gradients, tissue geometry, and directional delivery of soluble and extracellular signals all contribute to organ-selective vulnerability (15). In this context, the gut is unlikely to be merely a passive bystander. Rather, it should be understood as a selectively fragile and potentially amplifying organ whose barrier dysfunction helps explain why local intestinal instability may acquire systemic significance. This point is especially important within a source-stratified framework: in enterogenic sepsis, barrier collapse more often serves as an initiating interface for outward injury propagation, whereas in extraintestinal sepsis it more often emerges as an early remote target of circulatory, inflammatory, and neurohumoral disturbance (16).

Once intestinal barrier integrity is disrupted, the pathological process is no longer confined to the mucosal surface. Systemic inflammation, ischemia–reperfusion injury, endothelial damage, and oxidative stress can destabilize epithelial continuity and increase intestinal permeability, thereby weakening the separation between luminal contents and the host internal compartment (17, 18). Barrier loosening impairs epithelial defense and repair, reduces resistance to mechanical and inflammatory stress, and permits continued leakage of harmful luminal components and non-microbial injurious signals (19). In this setting, sepsis-associated acute gastrointestinal injury and paralytic ileus should no longer be regarded as transient local events, but as structural states in which mucosal injury becomes biologically capable of driving remote organ involvement.

Barrier remodeling also establishes a self-reinforcing pathological loop. Increased permeability promotes epithelial injury, defective restitution, mucosal edema, and persistent dysmotility, while simultaneously creating conditions that favor dysbiosis, inflammatory amplification, and progressive loss of homeostatic regulation (20). Once this loop is established, the gut becomes not only more permeable to harmful molecular traffic but also less capable of restoring epithelial continuity, preserving absorptive function, and maintaining enteral tolerance. Accordingly, barrier failure in sepsis is not merely a downstream manifestation of systemic illness; it is a central mechanistic interface through which local intestinal instability is converted into sustained interorgan injury propagation (10).

Taken together, barrier failure and permeability remodeling provide the structural platform on which subsequent dysbiosis, microbial translocation, immune dysregulation, metabolic disturbance, and extracellular signal transfer are superimposed (21). This perspective helps explain why the gut occupies a unique position in sepsis-associated acute gastrointestinal injury and paralytic ileus: it is simultaneously a fragile epithelial surface, a selectively vulnerable vascular–immune interface, and a key gateway through which local injury may be translated into systemic organ dysfunction (7).

### 2.2 Dysbiosis, microbial translocation, and metabolite signaling

Dysbiosis, microbial translocation, and metabolite signaling constitute the microecological layer of gut–extraintestinal organ crosstalk in sepsis-associated acute gastrointestinal injury and paralytic ileus. The gut is not merely a vulnerable epithelial surface but also the body’s most densely colonized microbial interface, and this ecological burden gives intestinal instability a unique capacity to influence distant organs (6). In sepsis, microecological disruption should therefore not be interpreted as a secondary epiphenomenon. Rather, it represents a mechanistically active process through which altered host–microbiota interactions convert local intestinal disturbance into distributed interorgan signaling (21).

The intestinal microbiome is one of the most important intermediaries of this process (22). Systemic inflammation, ischemia–reperfusion injury, and endothelial barrier damage may first disrupt intestinal mucosal integrity and increase permeability. Barrier loosening then promotes opportunistic pathogen expansion, depletion of commensal organisms, and loss of microbial diversity (23). The resulting dysbiosis further activates local inflammation, aggravates epithelial injury, and impairs mucosal repair. In this way, a self-reinforcing loop of inflammation, barrier disruption, dysbiosis, and further inflammation is established, transforming sepsis-associated acute gastrointestinal injury and paralytic ileus from apparently localized gastrointestinal manifestations into sustained biological drivers of systemic deterioration (24).

Once barrier integrity is impaired, the pathological process is no longer restricted to the intestinal lumen. Gut-derived bacteria, lipopolysaccharide, bacterial DNA, fungal components, and other microbe-associated molecules may cross the mucosa and gain access to the portal circulation, lymphatic system, and systemic bloodstream, thereby affecting distant organs such as the lung, liver, kidney, and brain (21). However, microbial translocation should not be viewed simply as one-way outward leakage from the intestine. In enterogenic sepsis, it more often represents an initiating route through which gut-derived pathological signals propagate toward extraintestinal organs (23). In extraintestinal sepsis, by contrast, pulmonary infection, broad-spectrum antimicrobial exposure, mechanical ventilation, altered nutritional delivery, and other organ-derived stresses may remodel the intestinal microbial community in return, thereby creating bidirectional dysregulation between the gut and distant organs under systemic infectious stress. This directional flexibility is one reason why the microbiological layer of sepsis cannot be reduced to a purely local intestinal event (12).

Beyond microbial composition and translocation alone, microbial metabolites represent another major route of interorgan communication. Short-chain fatty acids, bile acid-related metabolic networks, tryptophan-derived metabolites, and other microbiota-derived small molecules influence epithelial energy supply, tight-junction stability, immune-cell differentiation, and inflammatory signal transduction (24). During sepsis, depletion of short-chain fatty acid-producing bacteria, disruption of bile acid metabolism, and broader metabolic signaling disorder convert molecular systems that normally preserve barrier integrity and immune homeostasis into maladaptive or proinflammatory signaling networks. Through this shift, local microecological disturbance is translated into systemic organ injury not only by microbial escape, but also by altered metabolic information flow (25).

Importantly, these microecological changes do not act in isolation. Dysbiosis, microbial trafficking, and maladaptive metabolite signaling collectively reshape the downstream immune environment, thereby promoting excessive innate immune activation, imbalanced macrophage polarization, sustained neutrophil recruitment, and adaptive immune remodeling (25). Thus, in sepsis-associated acute gastrointestinal injury and paralytic ileus, microecological alteration should be understood not merely as a compositional change in the microbiota, but as a mechanistically active communication system through which the gut redistributes pathological signals to distant organs while simultaneously becoming more vulnerable to extraintestinal feedback injury. This perspective provides the basis for the subsequent discussion of immune dysregulation as the convergent downstream core of organ crosstalk (12).

### 2.3 Immune dysregulation and inflammatory signal propagation

Immune dysregulation and inflammatory signal propagation constitute a central immunological core of gut–extraintestinal organ crosstalk in sepsis-associated acute gastrointestinal injury and paralytic ileus (26). Regardless of whether the primary infectious focus is intestinal or extraintestinal, sepsis is initially characterized by the sustained release of pathogen-associated molecular patterns, damage-associated molecular patterns, inflammatory cytokines, and endotoxin-related signals into the circulation (27). This early inflammatory surge promotes endothelial activation, glycocalyx shedding, adhesion molecule upregulation, and coagulation imbalance, while the intestinal mucosa—because of its high perfusion sensitivity and barrier dependence—often becomes one of the earliest tissues to experience compromised integrity and functional decline. In this context, the gut is not merely a locally injured organ, but also a permissive interface through which inflammatory signals can be amplified and redistributed toward distant organs (28).

This immunological propagation is closely intertwined with microecological and metabolic alterations. Beyond changes in microbial composition alone, microbiota-derived metabolites such as short-chain fatty acids, bile acid-related metabolic networks, and tryptophan-derived molecules influence immune-cell differentiation, epithelial stability, and inflammatory signal transduction (12). During sepsis, depletion of short-chain fatty acid-producing bacteria, disruption of bile acid metabolism, and broader metabolic signaling disorder convert regulatory networks that normally preserve immune and barrier homeostasis into maladaptive or proinflammatory systems. As a result, local microecological disturbance is translated into systemic immune activation and remote organ injury (24).

Although different organ axes may manifest distinct biological and clinical phenotypes, their downstream immune pathways show substantial convergence. Dysbiosis and barrier disruption can jointly drive excessive innate immune activation, promote imbalanced macrophage polarization, sustain neutrophil recruitment, and reshape adaptive immunity (29). As sepsis progresses, this hyperinflammatory state often coexists with immunosuppression, leading to the simultaneous failure of pathogen clearance and aggravation of tissue injury (30). Accordingly, microecological alteration, barrier dysfunction, and immune dysregulation should not be viewed as parallel and independent processes, but rather as interdependent components of a shared immunological core underlying organ crosstalk.

Importantly, inflammatory signal propagation is not limited to soluble mediators alone. Resident macrophages, dendritic cells, endothelial cells, and enteric glial cells in the gut and extraintestinal organs are capable of sensing pathogen-associated cues, damage-associated signals, cytokines, and metabolic abnormalities originating from distant sites, and rapidly translating these inputs into local inflammation, permeability changes, and tissue injury responses (27). In this way, distant organs do not function merely as passive recipients of systemic inflammation, but as active participants in the reconstruction and amplification of the injury network. Once this circuit is established, it becomes difficult to terminate spontaneously because it remains embedded within persistent inflammation, immune imbalance, endothelial dysfunction, and interacting metabolic and microcirculatory disturbances. Consequently, acute gastrointestinal injury, paralytic ileus, and distant organ dysfunction may display both temporal persistence and spatially coordinated multiorgan deterioration (7).

Taken together, immune dysregulation in sepsis-associated acute gastrointestinal injury and paralytic ileus should be understood not simply as an increase in inflammatory burden, but as a distributed signaling process that links the gut to distant organs through convergent innate, adaptive, endothelial, and metabolic pathways (26). This perspective helps explain why gut-centered injury may evolve from an early localized disturbance into a sustained network of interorgan dysfunction (8).

### 2.4 Microcirculatory failure and perfusion mismatch

Microcirculatory failure and perfusion mismatch represent a central hemodynamic layer of gut–extraintestinal organ crosstalk in sepsis-associated acute gastrointestinal injury and paralytic ileus (31). Regardless of whether the primary infectious focus is intestinal or extraintestinal, sepsis is characterized early by the sustained release of pathogen-associated molecular patterns, damage-associated molecular patterns, inflammatory cytokines, and endotoxin-related signals into the circulation, thereby promoting endothelial activation, glycocalyx shedding, adhesion molecule upregulation, and coagulation imbalance (32). The resulting pathophysiological state is marked by the coexistence of microcirculatory hypoperfusion, tissue hypoxia, and capillary leakage, with the intestinal mucosa often among the earliest tissues to experience compromised perfusion. Because the intestinal villus tip is highly sensitive to hypoperfusion and hypoxia, and because epithelial renewal and barrier maintenance are energetically demanding, the gut readily develops perfusion imbalance, barrier instability, absorptive dysfunction, dysmotility, and feeding intolerance under systemic circulatory stress (33).

This vulnerability is further amplified when extraintestinal organ dysfunction perturbs regional blood flow and venous return. In the lung–gut axis, pulmonary infection, hypoxemia, acute lung injury or acute respiratory distress syndrome, and ventilation-associated biotrauma may redistribute visceral blood flow, exposing the villus tip to early ischemia and energetic stress while simultaneously aggravating endothelial activation and microcirculatory dysfunction (8). In the kidney–gut axis, acute kidney injury contributes to elevated venous pressure, interstitial fluid overload, endothelial dysfunction, and reduced mucosal perfusion, thereby directly promoting bowel-wall edema, luminal distension, and motility impairment (34). In the heart–gut axis, myocardial depression, low cardiac output, blood-flow redistribution, and venous congestion jointly reduce splanchnic perfusion and intensify bowel-wall congestion, edema, and barrier loosening. Although the proximal triggers differ across organs, these hemodynamic insults converge on a shared gastrointestinal phenotype characterized by mucosal hypoperfusion, edema, dysmotility, impaired enteral tolerance, and progression toward acute gastrointestinal injury or paralytic ileus (35).

Importantly, microcirculatory failure should not be interpreted as a purely passive ischemic process. Once perfusion mismatch is established, it interacts with barrier injury, microbial dysregulation, inflammatory propagation, and metabolic stress to sustain a self-reinforcing injury circuit (36). Impaired perfusion weakens epithelial integrity and repair capacity, venous congestion amplifies bowel-wall edema and diffusion distance, and endothelial dysfunction facilitates permeability increase and local immune disequilibrium. As a result, the gut may shift from an early vulnerable target of circulatory disturbance to a secondary amplifier of systemic inflammation and multiorgan deterioration. Even after the initial infectious source is controlled, pre-existing endothelial dysfunction, persistent microcirculatory impairment, and their interaction with immune and metabolic abnormalities may continue to sustain organ injury, thereby contributing to the temporal persistence and spatial coordination of multiorgan dysfunction in sepsis-associated acute gastrointestinal injury and paralytic ileus (7).

### 2.5 Mitochondrial dysfunction and immunometabolic reprogramming

Mitochondrial dysfunction and immunometabolic reprogramming constitute another major mechanistic layer of gut–extraintestinal organ crosstalk in sepsis-associated acute gastrointestinal injury and paralytic ileus (37). Compared with many other organs, the gut displays pronounced metabolic vulnerability during sepsis because the intestinal epithelium undergoes rapid turnover, barrier maintenance is highly energy-dependent, and the villus tip is particularly sensitive to hypoperfusion and hypoxia (38). Under septic stress, these features render the gut highly susceptible to energetic failure, oxidative injury, and defective epithelial renewal, thereby facilitating the early development of barrier instability, dysmotility, and feeding intolerance. In this context, the gut may shift from a metabolically vulnerable target to a secondary amplifier of systemic inflammation and multiorgan dysfunction (39).

Beyond microbial composition alone, microbiota-derived metabolites play a central role in this metabolic layer of organ crosstalk. Short-chain fatty acids, bile acid-related metabolic networks, tryptophan-derived metabolites, and other microbial small molecules influence epithelial energy supply, tight-junction stability, immune-cell differentiation, and inflammatory signal transduction (24). During sepsis, depletion of short-chain fatty acid-producing bacteria, disruption of bile acid metabolism, and broader metabolic signaling disorder convert molecular networks that normally preserve barrier integrity and immune homeostasis into maladaptive or proinflammatory signaling systems. As a result, local microecological disturbance is translated into systemic organ injury not only through microbial translocation and inflammatory amplification, but also through disordered interorgan metabolic communication (12).

These metabolic disturbances are further reinforced across specific organ axes. In the gut–kidney axis, dysbiosis-related metabolite changes and accumulation of uremic toxins aggravate renal inflammation and energy imbalance, while kidney dysfunction feeds back through toxin recirculation and immune-metabolic disequilibrium to worsen bowel-wall injury (40). In the gut–liver axis, gut-derived inflammatory signals induce hepatic metabolic reprogramming, and dysregulated bile acid metabolism further destabilizes the intestinal barrier and microbial ecosystem (39). In the gut–heart axis, gut-derived inflammatory mediators and metabolic imbalance promote cardiomyocyte inflammation, mitochondrial dysfunction, and energy metabolic derangement, while depletion of protective metabolites weakens myocardial tolerance to inflammatory and oxidative stress. Thus, metabolic injury should not be regarded as a secondary accompaniment to inflammation, but as an active integrative mechanism through which gut instability is linked to distant organ dysfunction (41).

Importantly, this metabolic layer also helps explain why organ injury in sepsis often persists even after the initial infectious source has been controlled. Once established, the injury circuit may remain embedded within persistent inflammation, microcirculatory dysfunction, immune imbalance, and mitochondrial damage (38). Pre-existing endothelial dysfunction, mitochondrial injury, and altered immune-cell composition may continue to sustain organ failure and prevent full recovery of epithelial, vascular, and metabolic homeostasis. Consequently, sepsis-associated acute gastrointestinal injury, paralytic ileus, and distant organ dysfunction may show both temporal persistence and spatially coordinated multiorgan deterioration. Taken together, mitochondrial dysfunction and immunometabolic reprogramming provide a mechanistic basis for understanding how gut-centered injury evolves from an acute local disturbance into a sustained network of systemic organ failure (37).

### 2.6 Regulated cell death and its extracellular vesicle-mediated propagation

Organ crosstalk in sepsis is mediated not only by inflammatory molecules and microbial signals, but also by the interorgan propagation of cellular stress and injury responses. Resident macrophages, dendritic cells, endothelial cells, and enteric glial cells in the gut and extraintestinal organs are capable of sensing pathogen-associated signals, damage-associated signals, cytokines, and metabolic abnormalities originating from distant sites, and rapidly translating these inputs into local inflammation, permeability changes, and cellular injury programs (42). Accordingly, distant organs should not be regarded as passive recipients of systemic injury, but as active participants in the reconstruction and amplification of the septic injury network through local sensing systems (27).

In sepsis, cellular injury and death are characterized by the parallel activation of multiple pathways (43). Apoptosis, pyroptosis, necroptosis, and dysregulated autophagy do not operate in isolation; rather, they reinforce one another through inflammasome activation, membrane rupture, release of danger signals, and metabolic failure (44). Once cell death increases in one organ, additional damage-associated molecules and proinflammatory mediators are released, thereby promoting endothelial destabilization, immune cell recruitment, and parenchymal cell injury in other organs (45). This creates a domino-like cascade of cross-organ amplification, indicating that regulated cell death is not merely a terminal manifestation of tissue injury, but an active driver of continued organ crosstalk and persistent multiorgan dysfunction (42).

At present, it remains difficult to determine whether any single mode of cell injury or death predominates across all septic contexts (43). Depending on the time window, infection source, target organ, and host immune status, the dominant pathway may shift dynamically among apoptosis, pyroptosis, necroptosis, and autophagy imbalance (44). This context dependency suggests that regulated cell death in sepsis should be interpreted within a time-, source-, and organ-specific framework rather than through a single universally dominant mechanism. Such dynamic complexity may also help explain why many single-target interventions have not yet achieved stable clinical translation (28).

### 2.7 Extracellular vesicles as interorgan signal carriers

Extracellular vesicles represent a shared communication layer within gut–extraintestinal organ crosstalk in sepsis-associated acute gastrointestinal injury and paralytic ileus (46). Released from activated, stressed, or dying cells, EVs carry proteins, lipids, DNA, RNA, and other bioactive cargoes, thereby transmitting pathological signals across cells, tissues, and distant organs (47). In sepsis, they are increasingly recognized as important carriers linking local tissue injury to systemic inflammation, vascular dysfunction, organ remodeling, and persistence of multiorgan failure. Accordingly, EVs should not be regarded as an additional organ-specific topic, but rather as an integrating mechanism through which gut-centered and extraintestinal pathological signals are redistributed across the interorgan network (48).

From a clinical perspective, circulating EV abundance and EV cargo profiles have already been associated with sepsis severity, organ dysfunction, and prognosis (49). Plasma exosome levels increase across the spectrum from sepsis to septic shock and correlate with SOFA scores as well as short-term and longer-term mortality (48). In parallel, endothelium-derived EVs expressing adhesion-related markers such as platelet/endothelial cell adhesion molecule-1 (PECAM-1) and vascular endothelial cadherin (VE-cadherin) reflect increased endothelial permeability and disease severity, supporting the biological relevance of EVs beyond purely experimental settings. These observations suggest that EVs may eventually contribute to biomarker development for sepsis stratification, vascular injury assessment, and organ-specific risk evaluation (46).

Within the gut-centered mechanistic framework proposed in this review, EVs provide a plausible bridge linking barrier failure, immune dysregulation, endothelial injury, coagulopathy, metabolic disturbance, and regulated cell-death propagation (47). At the immune level, EVs transfer miRNAs, inflammatory mediators, and danger-associated signals that reshape macrophage, neutrophil, and endothelial responses, thereby amplifying or sustaining inflammation (48). These cargos may include molecules directly relevant to regulated cell-death propagation, such as inflammasome-related components including ASC, danger-associated mediators such as HMGB1, and specific miRNAs that reinforce apoptotic, pyroptotic, and other inflammatory death-signaling cascades across organs. In this way, EV-mediated communication helps mechanistically connect regulated cell death to distributed organ injury amplification rather than leaving these two processes as parallel phenomena. At the vascular and coagulation level, EVs contribute to endothelial activation, barrier hyperpermeability, and tissue factor-related procoagulant activity, converting local infection signals into systemic microcirculatory dysfunction and immunothrombosis (46). At the metabolic and cell-fate level, EV cargoes containing mitochondrial components, lipids, and nucleic acids may participate in metabolic reprogramming, propagation of regulated cell death, and persistence of organ failure. Taken together, EVs function as an important mechanistic link through which diverse septic insults are integrated into a unified network of interorgan injury amplification, with downstream manifestations including barrier failure, motility impairment, feeding intolerance, and progression of sepsis-associated acute gastrointestinal injury and paralytic ileus (50).

The significance of EVs also lies in their ability to mediate both short-range and long-range communication. Within local tissues, EVs released by injured epithelial cells, immune cells, and endothelial cells can rapidly alter the inflammatory phenotype, barrier status, and repair capacity of neighboring cells (50). At a broader scale, EVs can travel through the bloodstream, lymphatics, and across biological barriers, thereby delivering pathological information from the gut or distant organs to the lung, brain, liver, kidney, and other targets. In addition, bacterial EVs released by the gut microbiota may participate in host–microbiota and gut–distal organ communication. Experimental evidence showing that septic plasma EVs can cross the blood–brain barrier and trigger microglial inflammatory responses further supports the role of EVs in the transition from local pathology to systemic interorgan signaling in sepsis (51).

**Figure 2:**
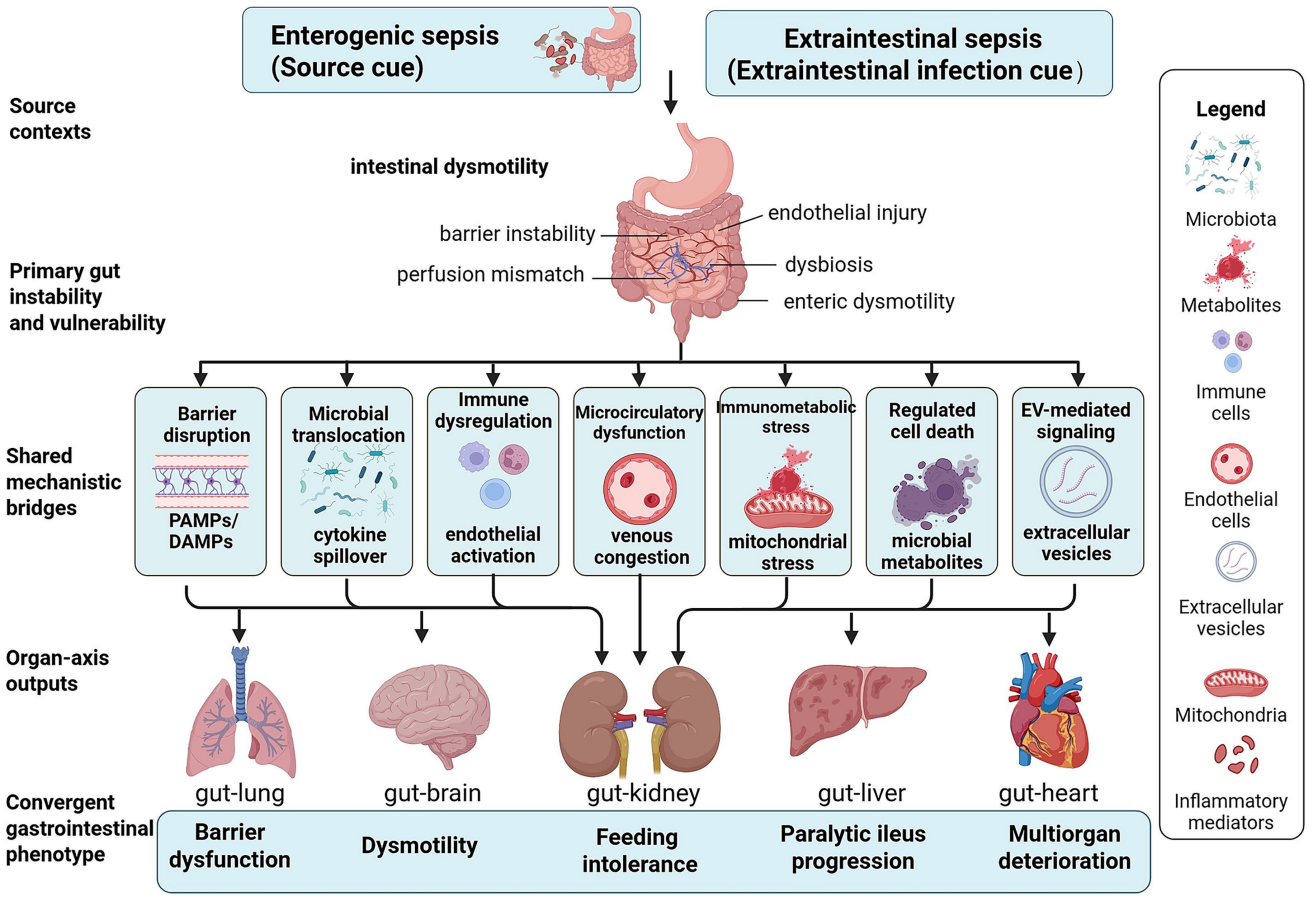
Shared gut-centered mechanistic framework linking source context to organ-axis outputs in sepsis-associated acute gastrointestinal injury and paralytic ileus. This figure summarizes the shared gut-centered mechanistic framework underlying organ crosstalk in sepsis-associated acute gastrointestinal injury and paralytic ileus. Enterogenic and extraintestinal septic contexts converge on a state of primary gut instability and vulnerability characterized by barrier instability, perfusion mismatch, endothelial injury, dysbiosis, and enteric dysmotility. These alterations are translated into interorgan injury propagation through several mechanistic bridges, including barrier disruption, microbial translocation, immune dysregulation, microcirculatory dysfunction, immunometabolic stress, regulated cell death, and extracellular vesicle-mediated signaling. The downstream outputs involve the gut–lung, gut–brain, gut–kidney, gut–liver, and gut–heart axes, which ultimately converge on barrier dysfunction, dysmotility, feeding intolerance, paralytic ileus progression, and multiorgan deterioration. EV, extracellular vesicle; PAMPs, pathogen-associated molecular patterns; DAMPs, damage-associated molecular patterns.

Taken together, these interconnected layers define a shared gut-centered mechanistic framework linking source context, primary intestinal instability, mechanistic bridges, and distal organ-axis outputs; this framework is illustrated in Figure 2. A simplified source-stratified overview of the major directional organ-axis patterns in enterogenic and extraintestinal sepsis is summarized in Table 1.

**Table 1:** Simplified source-stratified overview of gut–extraintestinal organ crosstalk in sepsis-associated acute gastrointestinal injury and paralytic ileus.

Section	Core concept	Main implication	References
A. Shared mechanistic framework	Barrier failure, dysbiosis, immune dysregulation, microcirculatory dysfunction, metabolic disturbance, regulated cell death, and extracellular signaling are mechanistically coupled.	Gut instability may both reflect and propagate multiorgan dysfunction.	(5, 6, 26, 31, 37, 42, 46)
B. Enterogenic sepsis: gut-to-extraintestinal organs	In enterogenic sepsis, the gut more often acts as an early driver of outward injury propagation.	Gut-derived signals contribute to secondary injury in distant organs.	(10, 16, 21)
Lung	Gut-derived inflammatory and microbial signals amplify pulmonary injury.	Acute lung injury and respiratory dysfunction may worsen.	(8, 21, 24)
Brain	Gut-derived inflammatory and metabolic signals promote neuroinflammation.	Encephalopathy, delirium, and cognitive dysfunction may develop.	(12, 26, 51)
Kidney	Gut-derived endotoxin and inflammatory mediators contribute to renal injury.	Septic acute kidney injury may be aggravated.	(21, 40, 42)
Liver	Portal delivery of gut-derived signals activates hepatic immune responses.	Bidirectional gut–liver injury amplification may occur.	(12, 21, 24)
Heart	Gut-derived inflammatory and metabolic stress may impair myocardial function.	Circulatory instability and septic cardiomyopathy may worsen.	(40, 41, 50)
C. Extraintestinal sepsis: extraintestinal organs-to-gut	In extraintestinal sepsis, the gut more often emerges as a vulnerable remote target.	Remote organ dysfunction converges on secondary gastrointestinal injury.	(7, 31, 40)
Lung	Hypoxemia, ventilation, and treatment-related exposures impair gut perfusion and motility.	Barrier injury, feeding intolerance, and ileus may develop.	(8, 31, 35)
Brain	Central-autonomic and neuroendocrine dysregulation suppress gastrointestinal function.	Dysmotility and enteral intolerance may worsen.	(7, 26, 28)
Kidney	Venous congestion, toxin retention, and metabolic stress damage the gut.	Bowel-wall edema, barrier dysfunction, and secondary AGI may occur.	(31, 34, 40)
Liver	Impaired detoxification and bile acid dysregulation destabilize gut homeostasis.	Dysbiosis, barrier instability, and paralytic ileus may be aggravated.	(12, 24, 40)
Heart	Low cardiac output and venous congestion reduce splanchnic perfusion.	Hypoperfusion-related barrier failure and dysmotility may develop.	(31, 35, 41)

This simplified table presents the main source-stratified framework of gut–extraintestinal organ crosstalk in sepsis-associated acute gastrointestinal injury and paralytic ileus. In enterogenic sepsis, the gut more often acts as an early driver of outward injury propagation, whereas in extraintestinal sepsis it more often emerges as a vulnerable remote target. Despite these different starting points, the downstream gastrointestinal phenotype remains broadly convergent, characterized by barrier disruption, dysmotility, feeding intolerance, and progression toward paralytic ileus.

AGI, acute gastrointestinal injury.

## 3 Organ-axis manifestations under the source-stratified framework

### 3.1 Enterogenic sepsis: gut-origin outward injury propagation

In enterogenic sepsis, the gut more often functions as an early driver of outward injury propagation rather than merely a downstream damaged organ (52). It simultaneously serves as the site of infectious onset, the interface of barrier failure, and a major hub of inflammatory, microbial, and metabolic signal amplification (53). Once mucosal ischemia, permeability increase, dysbiosis, and leakage of microbial or non-microbial injurious factors occur, these disturbances may spread through the portal circulation, mesenteric lymphatics, and systemic bloodstream, thereby promoting secondary injury in distant organs including the lung, brain, kidney, liver, and heart. Accordingly, enterogenic sepsis provides the clearest pathobiological context in which gut instability precedes and drives outward organ injury propagation. The following subsections summarize how this gut-originating injury pattern is expressed across the major extraintestinal organ axes.

### 3.2 Pulmonary injury as a downstream target of gut-origin signal escape

In enterogenic sepsis, the gut acts not only as the site of local barrier collapse but also as an initiating driver of downstream pulmonary injury, so that worsening acute gastrointestinal injury or paralytic ileus is often accompanied by increased risk of acute respiratory distress syndrome, deteriorating oxygenation, and escalating respiratory support (54). The core mechanistic chain involves barrier breakdown, portal and lymphatic escape of gut-derived microbial products and inflammatory mediators, systemic immune activation, and subsequent injury to the pulmonary endothelium and alveolar epithelial barrier (55). In parallel, neutrophil recruitment, capillary leakage, and alveolar inflammation intensify, thereby promoting acute lung injury and respiratory dysfunction (56). Beyond this shared inflammatory cascade, the gut–lung axis includes several relatively specific amplifiers, including loss of beneficial microbial metabolites, increased neutrophil extracellular trap formation, dysregulated macrophage polarization, Th17/Treg imbalance, and persistent stimulation of pulmonary innate immunity by gut-derived microbial components (57). These changes indicate that lung injury in enterogenic sepsis is not simply a passive consequence of systemic inflammation, but a secondary organ disorder shaped by reciprocal interactions among immune disequilibrium, barrier collapse, and microbial dysregulation. Pulmonary injury may in turn aggravate intestinal barrier instability, thereby creating a bidirectional positive-feedback loop (58, 59). What makes this route clinically important is not only the coexistence of lung injury and intestinal dysfunction, but their convergence on a common gastrointestinal phenotype of barrier destabilization, enteric hypomotility, reduced enteral tolerance, increased gastric residual volume, abdominal distension, and progression toward paralytic ileus.

### 3.3 Neuroinflammatory vulnerability under gut-origin stress signaling

In enterogenic sepsis, the gut acts as an initiating source of inflammatory, metabolic, and neuroimmune stress that may extend beyond the intestine and contribute to sepsis-associated encephalopathy, delirium, and long-term cognitive decline (60). These neurological manifestations are rarely explained by primary central nervous system infection alone and are more plausibly driven by the combined effects of peripheral inflammation and gut–brain crosstalk (61). The mechanistic chain involves several parallel routes. Gut-derived inflammatory mediators, endotoxin-related signals, and metabolic disturbances may increase blood–brain barrier permeability, activate microglia, and initiate neuroinflammation (62). At the same time, vagal afferent signaling, hypothalamic–pituitary–adrenal axis activation, and alterations in serotonin and tryptophan metabolism translate intestinal stress into central nervous system dysfunction, while disruption of neuroimmune coupling further weakens central anti-inflammatory restraint and facilitates the transition from gut-derived stress signaling to sustained neuroinflammation (63). Sepsis-related dysbiosis and depletion of protective metabolites such as short-chain fatty acids further weaken neuroimmune homeostasis, thereby facilitating progression from transient dysfunction toward persistent cognitive injury (64). These observations indicate that the gut–brain axis is not a one-way inflammatory conduit but a bidirectional network integrating neural, endocrine, immune, and metabolic signaling. The final intestinal expression of this network remains broadly convergent, with barrier dysfunction, dysmotility, feeding intolerance, and progression toward paralytic ileus as the dominant downstream phenotype.

### 3.4 Renal injury within the gut-origin inflammatory–metabolic loop

In enterogenic sepsis, the gut functions as an initiating source of outward inflammatory and metabolic spillover, and the kidney therefore emerges as a major downstream target of intestinal instability (65). Intestinal barrier failure, paralytic ileus, and sustained inflammation commonly coexist with acute kidney injury and are associated with difficult fluid management, reduced toxin clearance, and worse outcomes (66). The mechanistic chain centers on the fact that gut-derived endotoxin, microbial components, and inflammatory mediators entering the circulation can induce renal microvascular endothelial injury, tubular epithelial stress, and local immune amplification (67). Meanwhile, dysbiosis-related changes in microbial metabolites and accumulation of uremic toxins further aggravate renal inflammation and energy imbalance (68). Once kidney dysfunction develops, fluid retention, venous congestion, toxin recirculation, and immune disequilibrium may secondarily worsen bowel-wall edema and barrier injury, thereby giving the gut–kidney axis a clearly self-reinforcing character. Thus, the gut–kidney axis in enterogenic sepsis should be understood as a reciprocal circuit in which gut-derived inflammatory and metabolic stress promotes renal injury, whereas kidney dysfunction feeds back to aggravate bowel-wall edema, barrier failure, dysmotility, and feeding intolerance. In practical clinical terms, this sequence links toxin retention and venous-congestive bowel-wall injury to abdominal distension, reduced feeding tolerance, and an ileus-prone gastrointestinal phenotype.

### 3.5 Portal relay and hepatic buffering failure in gut-origin sepsis

In enterogenic sepsis, the liver occupies the most immediate downstream position after gut instability because portal drainage places hepatic immune buffering directly in the path of gut-derived injurious signals (69). Clinically, intestinal barrier disruption is often accompanied by hepatic dysfunction, cholestatic changes, and inflammatory amplification, indicating that the liver acts both as the first defensive checkpoint and as a highly exposed secondary target organ (70). Its core mechanistic chain depends mainly on the portal pathway and the intrinsic hepatic immune system. Gut-derived pathogen-associated and damage-associated signals, microbial products, and inflammatory mediators enter the portal circulation and are recognized by Kupffer cells, liver sinusoidal endothelial cells, and hepatocytes, thereby triggering cytokine release, acute-phase responses, metabolic reprogramming, and portal danger-signal sensing that weakens hepatic immune buffering and predisposes to bile acid signaling disequilibrium (71). When this defensive response becomes dysregulated, the liver’s ability to clear bacteria, detoxify injurious molecules, and regulate bile acid metabolism declines, which in turn destabilizes the intestinal barrier, reshapes the microbiota, and drives inflammatory spillback toward the gut (72). The gut–liver axis is therefore not a simple upstream–downstream route but a bidirectional disequilibrium network sustained by portal inflammatory inflow, hepatic immune buffering, and bile acid–microbiota dysregulation. Failed hepatic compensation may feed back to aggravate barrier injury, enteral intolerance, and progression toward paralytic ileus (73). Mechanistically, portal inflammatory loading and bile acid signaling disequilibrium promote microbiota instability and defective mucosal repair, which clinically manifest as worsening enteral intolerance, delayed gastrointestinal transit, and aggravation of paralytic ileus.

### 3.6 Myocardial suppression under gut-derived inflammatory and metabolic stress

In enterogenic sepsis, the heart should be viewed as a downstream target of gut-derived inflammatory, microbial, and metabolic stress rather than as a recipient of uniformly distributed systemic injury alone. Clinical and multi-omics evidence suggests that gut microbiota dysbiosis is associated with sepsis-induced cardiomyopathy, elevated lactate, and poor short-term outcomes, indicating that the heart is also an important target of outward propagation from intestinal instability (74). The core mechanism involves gut-derived endotoxin and inflammatory mediators entering the circulation and promoting cardiomyocyte inflammation, mitochondrial dysfunction, and energy metabolic derangement, while endothelial injury and microcirculatory impairment reduce myocardial perfusion, diminish effective forward flow, and lower splanchnic perfusion reserve, thereby creating a hemodynamic background that feeds back to aggravate intestinal ischemia-prone barrier failure and dysmotility (75). In parallel, microbial metabolic imbalance and depletion of protective metabolites may weaken myocardial tolerance to inflammatory and oxidative stress, ultimately manifesting as impaired contractility and circulatory instability (76). Thus, the critical issue in the gut–heart axis is not simply that inflammation reaches the myocardium, but that gut-derived inflammatory, metabolic, microbial, and microcirculatory stress collectively create a permissive environment for septic myocardial suppression. The downstream intestinal result is a source-linked pattern of barrier failure, dysmotility, feeding intolerance, and progression of sepsis-associated acute gastrointestinal injury and paralytic ileus (77). In bedside terms, myocardial suppression and reduced splanchnic perfusion reserve favor intestinal hypoperfusion and bowel-wall edema, which may present as worsening feeding intolerance, decreased bowel motility, and a more severe ileus-prone phenotype.

### 3.7 Summary of gut-to-extraintestinal organ crosstalk in enterogenic sepsis

Taken together, the lung, brain, kidney, liver, and heart may all function as major remote targets of outward injury propagation once the gut becomes unstable in enterogenic sepsis (78). In this setting, barrier failure, dysbiosis, microbial and non-microbial signal escape, inflammatory amplification, metabolic disturbance, and microcirculatory stress are mechanistically intertwined rather than sequentially isolated. Importantly, this outward spread is accompanied by progressive gastrointestinal deterioration, manifested by worsening barrier dysfunction, dysmotility, feeding intolerance, and evolution toward paralytic ileus. These observations support a gut-centered model in which local intestinal injury and systemic organ propagation are dynamically coupled (79).

### 3.8 Extraintestinal sepsis: convergent remote injury projected onto the gut

When sepsis originates from extraintestinal organs such as the lung, brain, kidney, liver, or heart, the gut more often emerges as a vulnerable remote target rather than the initial driver of injury (80). This vulnerability reflects the unique dependence of the intestinal mucosa on adequate perfusion, oxygen delivery, neurohumoral regulation, epithelial renewal, immune surveillance, and microbial homeostasis. Once extraintestinal organ dysfunction disrupts circulatory, neuroendocrine, metabolic, or inflammatory networks, the gut readily develops perfusion mismatch, dysmotility, dysbiosis, and barrier collapse, thereby progressing toward secondary acute gastrointestinal injury and paralytic ileus. The following subsections summarize how distinct extraintestinal organs converge on the gut through different proximal routes while ultimately producing a shared gastrointestinal phenotype of worsening barrier dysfunction, motility failure, and enteral intolerance (81).

#### 3.8.1 Pulmonary insult projected onto the gut

In extraintestinal sepsis, the gut more often emerges as a vulnerable remote target onto which pulmonary hypoxemia, ventilation-related stress, and systemic inflammatory spillover are projected (82). Clinically, severe pulmonary inflammation, worsening oxygenation, and mechanical ventilation are frequently accompanied by abdominal distension, feeding intolerance, increased gastric residual volume, and intestinal paralysis, indicating that the gut is an important vulnerable remote target after lung injury (83). The major mechanistic chain involves four interconnected levels. First, pulmonary infection and hypoxemia lead to redistribution of visceral blood flow, causing the villus tip to experience early ischemia and energetic stress. Second, lung injury-related systemic inflammation and ventilation-associated biotrauma further damage the intestinal mucosal barrier through cytokine spillover, endothelial activation, and microcirculatory dysfunction (84). Third, prolonged mechanical ventilation, sedation, antibiotic exposure, and interruption of enteral nutrition jointly promote intestinal dysbiosis. Fourth, injury to tight junctions, mucus-layer thinning, and local immune disequilibrium transform the gut from a passive target into an inflammatory amplifier, thereby facilitating subsequent multiorgan deterioration (85). Thus, the critical issue in the lung–gut axis is not merely passive intestinal ischemia, but the combined effect of hypoxia, ventilation-related inflammation, microbial disruption, and barrier collapse in generating secondary acute gastrointestinal injury. The common intestinal outcome is mucosal barrier disruption, impaired motility, reduced enteral tolerance, and progression toward secondary paralytic ileus (86). Mechanistically, hypoxemia and ventilation-related stress promote villus ischemia, mucosal energetic failure, and barrier injury, which clinically manifest as increased gastric residual volume, feeding intolerance, abdominal distension, and progression toward secondary ileus.

#### 3.8.2 Brain-centered neurohumoral projection onto the gut

In extraintestinal sepsis, the gut more often functions as a vulnerable downstream effector organ onto which central neurohumoral disequilibrium and brain-centered inflammatory stress are projected (87). Clinically, sepsis-associated encephalopathy, delirium, and related forms of central dysfunction are often accompanied by impaired gastrointestinal motility and reduced enteral tolerance, suggesting that the gut is an important downstream target of brain-centered injury rather than merely a coincidental bystander (88). The mechanistic chain can be summarized at several interconnected levels. Central inflammation and sepsis-associated encephalopathy disrupt autonomic regulation, with sympathetic overactivation and vagal withdrawal suppressing gastrointestinal propulsion and predisposing to ileus, while sustained loss of vagal anti-inflammatory restraint favors mucosal immune disequilibrium and amplifies the transition from neurogenic dysmotility to barrier-compromising intestinal injury. Sustained hypothalamic–pituitary–adrenal axis activation and neuroendocrine stress contribute to splanchnic vasoconstriction, mucosal hypoperfusion, and impaired epithelial homeostasis (89). Neuroimmune disequilibrium and enteric glial or neural dysfunction amplify intestinal inflammatory responses, weaken barrier integrity, and disturb local immune balance, indicating that autonomic imbalance and sustained hypothalamic–pituitary–adrenal stress are translated into gut injury not only through hypoperfusion but also through impaired enteric glia–epithelial neuroimmune coupling. Reduced motility, delayed feeding, sedation exposure, and other treatment-related factors further reshape the microbiota, thereby aggravating dysbiosis, barrier failure, and enteral intolerance. Accordingly, in extraintestinal sepsis the brain–gut axis should not be understood primarily as a gut-to-brain inflammatory route, but rather as a top-down pathophysiological projection in which central dysfunction is translated into intestinal hypomotility, perfusion mismatch, and barrier injury. The downstream gastrointestinal phenotype remains one of dysmotility, barrier dysfunction, feeding intolerance, and progression toward paralytic ileus (90). In clinical terms, this top-down neurohumoral sequence may be expressed as decreased bowel motility, abdominal distension, reduced enteral tolerance, and an increased tendency toward paralytic ileus.

#### 3.8.3 Renal congestive and metabolic projection onto the gut

In extraintestinal sepsis, the gut becomes a vulnerable downstream target of renal congestive, metabolic, and inflammatory injury rather than a coincidental bystander of acute kidney dysfunction (91). Clinically, declining renal function is frequently accompanied by abdominal distension, bowel-wall edema, reduced feeding tolerance, and impaired intestinal barrier function, indicating that the gut is an important remote target of renal injury (92). The mechanistic chain reflects the fact that kidney injury is not merely loss of filtration, but a systemic disturbance involving fluid retention, elevated venous pressure, uremic toxin accumulation, acid–base imbalance, and amplification of inflammation. Venous congestion and interstitial fluid overload directly induce bowel-wall edema, luminal distension, and motility impairment. Uremic solutes and metabolic waste products can damage epithelial integrity and interfere with tight-junction organization (93). At the same time, kidney injury-related inflammation, oxidative stress, and endothelial dysfunction further reduce mucosal perfusion and disrupt local immune homeostasis. Renal injury also reshapes the microbiota, decreasing protective metabolites while increasing proinflammatory metabolic products, thereby establishing a vicious cycle of kidney injury, barrier breakdown, and renewed inflammation. Thus, intestinal injury in this axis should not be interpreted as a mere by-product of acute kidney injury, but as the remote projection of renal injury through circulatory, metabolic, and immune pathways. What ultimately emerges is a congestive and permeability-dominant gastrointestinal phenotype characterized by bowel-wall edema, barrier dysfunction, dysmotility, impaired enteral tolerance, and progression toward secondary acute gastrointestinal injury (94). Mechanistically, venous congestion and uremic-metabolic burden promote permeability increase and interstitial bowel-wall swelling, which clinically manifest as abdominal distension, reduced feeding tolerance, and progression of secondary acute gastrointestinal injury.

#### 3.8.4 Hepatic detoxification and bile-acid failure projected onto the gut

In extraintestinal sepsis, the gut becomes a vulnerable downstream target of hepatic detoxification failure, bile acid disequilibrium, and liver-centered immune dysregulation (95). Clinically, cholestasis, impaired detoxification, and hepatic immune disequilibrium are often accompanied by intestinal barrier disruption and microbiota instability, indicating that liver dysfunction substantially reshapes the intestinal environment (96). The major mechanistic chain centers on bile acid metabolism, detoxification failure, and disturbed immune homeostasis. Impaired hepatic synthesis and secretion of bile acids alter the luminal bile acid profile, directly affecting microbial composition, epithelial renewal, and mucosal barrier stability (97). When hepatic clearance of injurious molecules declines, a greater burden of inflammatory and microbial signals continuously acts on the gut, promoting local inflammation and permeability. In addition, disruption of immune regulation mediated by Kupffer cells, liver sinusoidal endothelial cells, and hepatocytes feeds back through the portal–gut signaling circuit and destabilizes intestinal mucosal immunity, thereby linking hepatic immune-buffering failure to persistent portal inflammatory loading, bile acid–microbiota disequilibrium, and defective epithelial renewal in the gut. Together, bile acid dysregulation, enhanced bacterial translocation, and defective mucosal repair render the gut a remote victim of liver dysfunction. Thus, intestinal injury in the liver–gut axis should not be reduced to a nonspecific digestive complication, but instead should be understood as the product of disturbed bile acid signaling, portal-circulatory dysregulation, impaired detoxification and immune surveillance, and altered microbial ecology. The resulting intestinal phenotype is one of barrier instability, microbiota-related enteral intolerance, and progression toward secondary paralytic ileus (98). Mechanistically, bile acid dysregulation and impaired detoxification promote dysbiosis, mucosal instability, and defective epithelial renewal, which clinically manifest as worsening enteral intolerance and aggravation of secondary paralytic ileus.

#### 3.8.5 Circulatory failure projected onto the gut

In extraintestinal sepsis, the gut more often emerges as a vulnerable remote target of circulatory failure, reduced forward flow, and venous congestion originating from myocardial depression (99). Low cardiac output, redistribution of blood flow, and venous congestion can directly cause intestinal mucosal hypoperfusion, bowel-wall edema, and motility suppression, thereby promoting acute gastrointestinal injury or paralytic ileus (100). The core mechanism can be summarized as follows. Myocardial depression and circulatory failure reduce splanchnic perfusion, while the intestinal villus is especially sensitive to ischemia because of its countercurrent exchange architecture. Simultaneously, elevated venous pressure and volume loading produce bowel-wall congestion, edema, and barrier loosening (101). When these hemodynamic insults are superimposed on sepsis-related inflammation, mitochondrial dysfunction, and microbial disequilibrium, the gut develops not only malabsorption and dysmotility but also greater susceptibility to translocation of bacterial products and secondary inflammatory amplification, because myocardial energetic failure and endothelial leakage further reduce effective splanchnic flow while intensifying bowel-wall interstitial edema (102, 103). In this sense, the heart–gut axis is driven by a triad of hypoperfusion, congestion, and inflammatory-metabolic dysregulation. The final common intestinal pattern is hypoperfusion-related barrier failure, edema-associated dysmotility, and worsening enteral intolerance (104). In clinical sequence, myocardial depression and venous congestion reduce intestinal perfusion, intensify bowel-wall edema, and ultimately present as impaired bowel sounds, abdominal distension, worsening feeding intolerance, and progression toward acute gastrointestinal injury or paralytic ileus.

#### 3.8.6 Summary of extraintestinal organ-to-gut crosstalk in extraintestinal sepsis

Taken together, in extraintestinal sepsis-associated secondary acute gastrointestinal injury and paralytic ileus, the gut is usually not the primary source organ but a remote target that is highly vulnerable to hypoxia, hypoperfusion, venous congestion, neuroendocrine disequilibrium, immune dysregulation, and metabolic disturbance (80). Although the proximal routes differ across the lung, brain, kidney, liver, and heart, these insults converge on a shared gastrointestinal phenotype characterized by barrier collapse, dysmotility, enteral intolerance, and progression toward paralytic ileus. These observations support the view that secondary gastrointestinal injury in sepsis is a convergent consequence of multiple extraintestinal organ-derived insults rather than a purely local digestive complication (81).

Representative evidence supporting gut-centered extracellular vesicle-mediated communication in sepsis-associated acute gastrointestinal injury and paralytic ileus is summarized in Table 2, with emphasis on directional relevance to the gut, source cells or organs, target sites, representative cargoes, and their proposed roles in interorgan signaling.

**Table 2:** Simplified overview of gut-centered extracellular vesicle-mediated communication in sepsis-associated acute gastrointestinal injury and paralytic ileus.

Directional relevance to the gut	Source cell/organ	Target cell/organ	Representative cargo or signal	Main implication	References
Local gut communication	Gut bacteria-derived EVs	Intestinal epithelial cells	Lipids, proteins, nucleic acids, and related EV cargos	Modulate the intestinal microenvironment and reshape epithelial barrier homeostasis.	(46, 47)
Systemic EV signals relevant to gut-centered injury propagation	Plasma exosomes from septic patients	Systemic organ injury phenotypes	Changes in exosome abundance and cargo	Reflect sepsis severity, organ dysfunction burden, and mortality risk.	(46, 48)
Systemic vascular EV signaling	Endothelium-derived EVs	Vascular endothelium	PECAM, VE-cadherin, and related adhesion molecules	Indicate endothelial permeability increase and support the vascular relevance of EV-mediated injury propagation.	(46, 49)
Long-range signaling potentially relevant to gut–brain crosstalk	Serum EVs after LPS challenge	CNS microglia and astrocytes	miR-155 and inflammatory cargos	Support the feasibility of EV-mediated transmission of systemic inflammatory signals to the brain.	(47, 51)
Gut-to-lung long-range communication	Gut microbiota/probiotics	Airway epithelium	Metabolites and EV-associated signals	Suggest that gut-derived microbial signaling may modulate distal airway inflammation.	(58, 59)

This simplified table summarizes representative evidence supporting gut-centered extracellular vesicle-mediated communication in sepsis-associated acute gastrointestinal injury and paralytic ileus. To maintain thematic focus in the main text, only evidence directly related to local gut signaling, gut-to-distal organ communication, or systemic extracellular vesicle pathways plausibly relevant to gut-centered organ crosstalk is retained. The expanded version with additional studies and detailed interpretation is provided in Supplementary Table 2.

## 4 Future directions and research challenges

Future research should be explicitly organized around the source-stratified framework proposed in this review. The first priority is to determine how the role of the gut shifts across septic contexts. In enterogenic sepsis, the key question is through which dominant routes gut-derived inflammatory, microbial, metabolic, vascular, and extracellular signals propagate toward distant organs. In extraintestinal sepsis, the corresponding question is through which organ-specific pathways lung-, brain-, kidney-, liver-, or heart-derived insults converge on the gut and trigger secondary acute gastrointestinal injury, with progression toward paralytic ileus in severe motility-dominant cases. The second priority is to define temporal hierarchy. It remains unclear whether the dominant mechanisms evolve from early perfusion mismatch, barrier destabilization, and inflammatory spillover toward later immune dysregulation, immunometabolic remodeling, extracellular vesicle-mediated communication, and regulated cell-death propagation. The third priority is to identify axis-specific decisive nodes, including cellular subsets, molecular mediators, and ligand–receptor interactions that are both biologically central and clinically actionable.

Future studies should also more explicitly align organ-axis mechanisms with monitorable biomarkers, clinically recognizable intermediate phenotypes, and source-stratified trial design. In the gut–brain axis, dynamic monitoring of intestinal fatty acid-binding protein, short-chain fatty acid-related metabolic signatures, and related neuroimmune indicators may help identify patients at risk of distant neurocognitive injury and enrich longitudinal studies of sepsis-associated encephalopathy. In the gut–kidney and heart–gut axes, bowel-wall edema, enteral intolerance, and perfusion-related variables may help define congestive- or hypoperfusion-dominant intermediate phenotypes and support hemodynamic gut-protective trial strategies. In the gut–liver axis, bile acid-related profiles, microbiota–bile acid interaction signals, and markers of impaired enteral tolerance may help capture patients with mucosal instability and paralytic ileus aggravation, thereby enabling mechanistically informed subgrouping. In enterogenic versus extraintestinal sepsis, clinical trials should move beyond treating sepsis-associated acute gastrointestinal injury as a uniform complication, because severe motility-dominant cases may progress toward paralytic ileus; instead, it should be approached as a source-dependent, time-sensitive, and organ-axis-defined syndrome, with enterogenic phenotypes being more suitable for barrier-protective or microbiota-modulating interventions and extraintestinal phenotypes requiring greater emphasis on perfusion-oriented and remote-organ-targeted mitigation strategies.

A major barrier to progress is the persistent gap between experimental models and clinical reality. Preclinical models differ substantially in infectious source, severity, hemodynamic context, and observation endpoints, limiting their ability to reproduce the heterogeneity of critically ill patients. Clinically, continuous monitoring of barrier integrity, mucosal perfusion, microbiota dynamics, neurohumoral disequilibrium, and organ-axis interactions remains inadequate, and many currently reported associations still lack causal closure. The most meaningful opportunities therefore lie not in further descriptive expansion alone, but in integrating source-stratified clinical cohorts with temporally resolved mechanistic platforms. Organoids and organ-on-chip systems may help reconstruct cross-organ microenvironments, whereas multi-omics, spatial transcriptomics, and single-cell ligand–receptor analyses may resolve the temporal and spatial heterogeneity of injury propagation. If linked to prospective phenotyping and longitudinal clinical sampling, these approaches may help identify dominant propagation routes, validate monitorable biomarkers, and define clinically actionable intervention windows for barrier protection, motility recovery, and improved enteral tolerance.

## 5 Conclusion

Sepsis-associated acute gastrointestinal injury should no longer be regarded as merely a localized gastrointestinal complication, and severe motility-dominant cases may progress toward paralytic ileus. Rather, they represent gastrointestinal manifestations of a broader interorgan injury network in which the gut assumes a source-dependent dual role. In enterogenic sepsis, the gut more often functions as an early driver of systemic propagation through barrier failure, dysbiosis, microbial and non-microbial signal escape, and inflammatory-metabolic amplification. In extraintestinal sepsis, the gut more often emerges as a vulnerable remote target of hypoperfusion, venous congestion, neurohumoral disequilibrium, immune dysregulation, and metabolic disturbance. Despite these different starting points, the downstream gastrointestinal phenotype is broadly convergent, characterized by barrier disruption, dysmotility, feeding intolerance, and progression toward paralytic ileus. Future studies and interventions should therefore move beyond descriptive organ-specific observations and instead prioritize source-based stratification, mechanism-to-phenotype alignment, and translationally actionable targets within a gut-centered framework of organ crosstalk.
